# The Occupational Risk of Influenza A (H1N1) Infection among Healthcare Personnel during the 2009 Pandemic: A Systematic Review and Meta-Analysis of Observational Studies

**DOI:** 10.1371/journal.pone.0162061

**Published:** 2016-08-31

**Authors:** Janna Lietz, Claudia Westermann, Albert Nienhaus, Anja Schablon

**Affiliations:** 1 Institute for Biostatistics and Social Welfare Matters, Hamburg, Germany; 2 Competence Centre for Epidemiology and Health Service Research in Nursing, Institute for Health Service Research in Dermatology and Nursing, University Medical Centre Hamburg-Eppendorf, Hamburg, Germany; 3 Department of Occupational Health Research, German Social Accident Insurance Institution for the Health and Welfare Services, Hamburg, Germany; University of Hong Kong, HONG KONG

## Abstract

**Introduction:**

The aim of this review was to record systematically and assess the published literature relating to the occupational risk of influenza A (H1N1) infection among healthcare personnel during the 2009 pandemic.

**Methods:**

The literature search was performed in June 2015. An update was carried out in May 2016. It was applied to the electronic databases EMBASE, MEDLINE, PsycINFO, PubMed, CINAHL and Google Scholar. The quality assessment was conducted with a tool using eight criteria. A meta-analysis was carried out to compute pooled effect estimates for influenza A (H1N1) infection.

**Results:**

A total of 26 studies were included in the review, 15 studies met the criteria for the meta-analysis. After a sensitivity analysis the pooled analysis showed a significantly increased odds for influenza A (H1N1) infection for healthcare personnel compared to controls/comparisons (OR = 2.08, 95% CI = 1.73 to 2.51). The pooled prevalence rate for healthcare personnel alone was 6.3%.

**Conclusions:**

This review corroborates the assumption that healthcare personnel were particularly at risk of influenza A (H1N1) infection during the 2009 pandemic. Healthcare facilities should intensify their focus on strategies to prevent infections among healthcare personnel, especially during the first period of pandemics.

## Introduction

In March 2009, infections with the novel influenza A (H1N1) virus were first reported in Mexico and the United States of America. The virus rapidly spread worldwide [1]. As a consequence, in June 2009, the World Health Organization (WHO) declared a global influenza pandemic [2]. A year later, in August 2010, the global pandemic was officially announced as ended [3]. Eventually, more than 214 countries and overseas territories or communities reported laboratory-confirmed cases of 2009 pandemic influenza A (H1N1), including 18,449 deaths [4]. Accordingly, the influenza A (H1N1) virus became a major public health problem around the world. During the 2009 influenza A (H1N1) pandemic, several epidemiological studies examined infection rates and risk factors leading to H1N1 infection among healthcare personnel (HCP) [5,6]. Due to close contact with infected patients, HCP are exposed to infectious microbes and therefore vulnerable to occupationally acquired infectious diseases such as influenza A (H1N1) [7]. In the United States of America the estimated annual death rate among HCP due to occupational infections was between nine and 42 deaths per million HCP [8]. However, the risk of influenza A (H1N1) infection for HCP was not definitively clarified. The present review therefore aimed to record systematically and assess the published literature relating to the occupational risk of influenza A (H1N1) infection among HCP during the 2009 pandemic. To examine whether HCP ran an increased risk of influenza A (H1N1) infection during the 2009 pandemic, a meta-analysis was performed.

## Methods

This systematic review was reported in line with the proposal for reporting Meta-analyses Of Observational Studies in Epidemiology (MOOSE checklist) [9].

The study protocol for this review was written on the basis of the Preferred Reporting Items for Systematic review and Meta-Analysis Protocols (PRISMA-P) statement [10]. It can be obtained from the corresponding author. The protocol was written in German and translated in English. It primarily documents the criteria for inclusion and contains a detailed description of applied research methods.

### Eligibility criteria

For the screening of identified studies several eligibility criteria were defined following the PECOS criteria [11]. Studies were included if the study population comprised healthcare professionals who had direct contact with patients and/or colleagues. It was specified that the occupation in healthcare had to involve exposure to infectious patients or materials. Studies with different or no occupational exposure were not considered. Moreover, studies were included if they used employees in (non-)healthcare without occupational exposure or reference data related to the general population from other studies as control/comparison group. Thereby the authors distinguish between population-based controls/comparisons (e.g. general population, non-healthcare professionals) and hospital-based controls/comparisons e.g. other healthcare professionals). Studies that did not report any valid control/comparison group or reference data were excluded. Outcome measures were laboratory-confirmed H1N1 infection, seroconversion or seropositivity. According to the Robert-Koch Institute (RKI), evidence of influenza A (H1N1) should be verified by one of the following direct or indirect testing methods: rapid influenza diagnostic test (RIDT), immuno-fluorescent antibody test (IFA), enzyme-linked immunosorbent assay (ELISA), reverse-transcription polymerase chain reaction (RT-PCR), haemagglutination inhibition test (HAI), microneutralisation test (MN) or viral isolation (VI) or culture (VC) [12]. Studies with no evidence of influenza A (H1N1) or with evidence of other types of influenza (B, C) and other subtypes of influenza A were excluded. Primarily, observational studies (e.g. cross-sectional studies, cohort studies and case-control studies) were included. Other study designs such as surveillance studies were also considered when the studies reported the total number of HCP, also stratified by hospital department and clinical occupation if possible. Articles were not limited to peer-reviewed publications. Content analyses, discussion papers, conference proceedings, theses and letters to the editor were also included. Publications that focused on the 2009 influenza A (H1N1) pandemic were included. Studies describing earlier pandemics, epidemics, outbreaks, or patients/residents in healthcare settings only were excluded. Therefore, articles relating to investigations between March 2009 and December 2010 were considered. Finally, there were no linguistic or geographical restrictions.

### Information sources

The authors JL, AS and research assistant EM developed the search strategy and conducted the search. It was applied to the electronic databases EMBASE, MEDLINE, PsycINFO, CINAHL (all via OVID), PubMed and Google Scholar. The authors searched for articles published between March 2009 and June 2015. The systematic database search was run in June 2015. An update of the literature search in May 2016 did not reveal any further relevant studies. In addition, reference lists of included studies and similar review articles were reviewed to identify further sources. The reference screening was conducted systematically by JL during the full-text screening of identified studies. In a few cases study authors were contacted via email to obtain additional information or publications.

### Search strategy

The following search terms and Medical Subject Headings (MeSH) were used to search all included databases:

- Influenza A H1N1 OR influenza virus A H1N1 OR influenza A virus, H1N1 subtype OR influenza virus A, subtype H1N1

AND

- Health care workers OR health personnel OR health care personnel

AND

- Occupational exposure OR occupational risk

AND

- Pandemic OR pandemic influenza OR pandemic* OR pandemics.

A detailed description of the search strategies of all included databases can be found in **S1 Appendix**.

### Study selection

Literature screening and eligibility assessment were performed independently by three reviewers (JL, AS and CW). The screening process was divided into two parts: title and abstract screening, and full-text screening. The authors developed a standardised screening instrument for both parts. If a study clearly met all predefined eligibility criteria (e.g. PECOS) it was included in the review. In all other cases the study was excluded. Disagreements between the two reviewers assessing the same paper were resolved by joint discussion. If no agreement could be reached, the third reviewer (AS or CW) performed a further literature screening and assessment of the study independently. Afterwards, uncertainties were clarified. The reviewers agreed on 87 out of 93 studies, that is 93%. No approval of the study by ethics committee was required as the authors performed a systematic review and meta-analysis of published literature.

### Data collection process

Data extraction from included studies was conducted independently by three reviewers (JL, AS and CW), with one author (JL) extracting the detailed data and a second author (CW) checking the extracted data for accuracy. The authors developed a data extraction form to collect information on the following aspects: 1) study characteristics (e.g. country, healthcare setting, study population, sample size and non-responder) and 2) results (e.g. diagnostic test, number and percentage of subjects infected with H1N1 virus as well as number and percentage of H1N1 infections related to occupation or working area). The extraction form comprised eighteen items. In case of uncertainty, a shared discussion took place. One author (JL) transferred the data to a table using Microsoft Excel 2013. If possible, missing data was calculated and added by JL. Some study authors were contacted to obtain more information on the described results or missing data. If a study has been published more than once, the authors considered only the report with the more detailed description of methods and results.

### Quality assessment

The quality of included studies was assessed independently by three authors (JL, AS and CW). Disputable cases were discussed. The risk of bias in particular studies was mainly considered using criteria for the assessment of methodological study quality. The developed assessment tool comprises fourteen items categorised in eight quality criteria (**Table 1**). Eleven items were taken from two standardised and well-validated checklists and modified [13–15]. Three were added by the authors. The items are expressed as questions (e.g. “Are the study design and sampling method appropriate for the research question?”). The answers are graded as yes (1 point), no (0 points) and unable to determine (0 points). Consequently, the study quality was assessed on a three-point scale as high (≥10 points), moderate (≥5 points) or low (≤4 points).

**Table 1:** Checklist for the quality assessment of influenza A (H1N1) studies among HCP.

Number	Criteria	Item
1	Study aim	Is the aim of the study clearly and precisely described?**
2	Study design	Are the study design and sampling method appropriate for the research question?*
3	Study population	Are the study subjects and the setting described in detail and similar to those of interest to you?*
4		Is the sampling frame appropriate?*
5		Is the sample size adequate?* (≥ 50 subjects per group)
6		Is the response rate adequate?* (≥ 60%)
7		Are the refusers described?*
8	Exposure	Are the results stratified by occupational group or exposure?***
9	Control group	Is a control group included in the study?***
10	Outcome	Are objective, suitable and standard diagnostic tests used for evidence of influenza A (H1N1) infection? If yes, which ones?* (e.g. RT-PCR: ≤ 38, HAI: ≥ 1:40, MN: ≥ 1:40)
11		Is the evidence of influenza A (H1N1) infection measured in an unbiased fashion? Is a confirmatory test performed?*
12	Analysis	Are the main findings of the study clearly described?**
13		Was there adequate adjustment for confounding in the analyses from which the main findings were drawn?**
14	Limitations	Were possible methodological limitations of the study discussed?***

Abbreviations: HAI: haemagglutination inhibition test, MN: microneutralisation test, RT-PCR: reverse-transcription polymerase chain reaction.

*Item adopted from [14,15].

**Item adopted from [13].

***Item added by the authors.

### Statistical analysis and data synthesis

Since an adequate number of studies were available, a meta-analysis was conducted by JL and CW. A study was involved in the meta-analysis if it included the following data for HCP (experimental) and non-HCP (control/comparison): cases (H1N1 pos.), non-cases (H1N1 neg.) and sample size (n). Seropositivity for influenza A (H1N1) was given if the HAI antibody titre was ≥ 1:40 or the RT-PCR value was ≤ 38 [12,16]. If multiple diagnostic tests were reported in the studies, the most recognised testing method was used to report the final estimate. In this case HAI and RT-PCR were used. Both tests are universally applicable, well validated and frequently used in studies investigating influenza A (H1N1). The purpose of the analysis was to compute combined effect estimates for influenza A (H1N1) infection among HCP, using the Mantel-Haenszel method for binary outcomes. Pooled prevalence ratios, odds ratios (OR) as effect estimates and 95% confidence intervals (95% CI) were calculated. In a subgroup analysis, studies were stratified by study quality (high and moderate). The presence of heterogeneity was tested using the χ² test, taking p < 0.10 as the level of significance. An I² test was performed to quantify the diversity between studies. With a sensitivity analysis, the impact of the single studies on the pooled effect estimate finding was verified by stepwise excluding studies from the meta-analysis and examining the estimate stability [17]. In the event of a clear difference in the combined estimate (OR) or I², the studies were excluded from further analyses. Possible publication bias due to study size was assessed graphically by means of a funnel plot. The bias is indicated by asymmetry in the plot. If applicable an Egger’s linear regression calculation was carried out to test the probability of publication bias by regression analysis [18]. The level of significance for asymmetry is taken as p < 0.10, the calculated intercept is given with a 90% confidence range. The meta-analysis was performed using Review Manager (RevMan 5.3).

## Results

### Study selection

**Fig 1:**
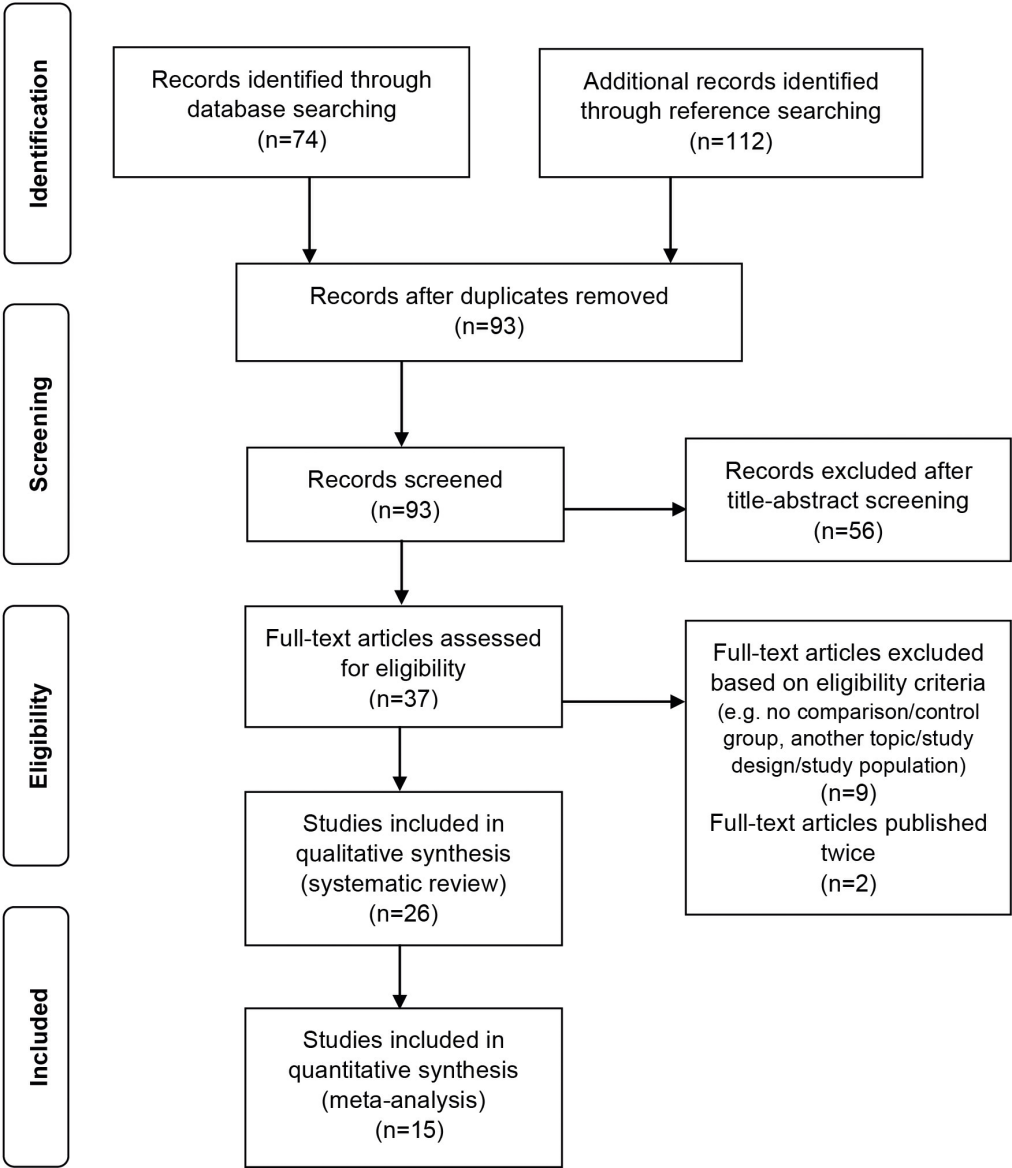
Study selection process for this systematic review (PRISMA flowchart).

The search of PubMed, MEDLINE, EMBASE, CINAHL, PsycINFO databases and Google Scholar provided a total of 74 citations. Through reference searching, 112 studies were found. After adjusting for duplicates, 93 studies remained. Of these, 56 studies were excluded after title- and abstract screening because they clearly did not meet the eligibility criteria. Of the remaining 37 studies that were subjected to full-text screening, nine did not fulfil the eligibility criteria and two had been published twice in different journals. Main reasons for exclusion from the review were a divergent study topic (e.g. influenza vaccination, personal protective equipment use and prevention programmes), a different study population (e.g. focus on influenza patients only), the absence of a control/comparison group or reference data and a different study design (e.g. review, intervention study, outbreak investigation and case study). A total of 26 studies were included in the review (**Fig 1**). They included 20 observational studies (fourteen cross-sectional studies and six cohort studies) and six other studies (three surveillance studies, two content analyses and one case-case control study). Of these, 15 studies were included in the meta-analysis. Reasons for exclusion of eleven studies [6,19–28] from the meta-analysis were missing data relating to the number of cases.

### Study characteristics

All 26 studies included in this review were published in English. Eight observational studies were based in Asia (Singapore, Korea, Japan, India, Taiwan and China; **Table 2**). Four studies each were carried out in North America (United States of America and Canada) and in Europe (Spain, Portugal, The Netherlands and Scotland). Three studies were based in Oceania (Australia and New Zealand) and one study in Central America (Mexico). Around 40% of the studies (n = 8) were carried out in general hospitals, five studies in acute care and five in tertiary care hospitals (emergency departments and influenza units), three studies in general practices and one study each in nursing homes, outpatient clinics and primary care medical centres. A variety of healthcare professionals were included in the studies as study subjects (e.g. physicians, general practitioners, nurses, nursing/doctors assistants, therapists, technicians, allied health/support and administrative staff). All studies performed standardised laboratory tests to confirm the evidence of influenza A (H1N1) infection among study participants. Most studies used the haemagglutination inhibition test (HAI; n = 17) or the reverse-transcription polymerase chain reaction method (RT-PCR; n = 10). Eight of the twenty studies (40%) completed a confirmatory test [16,23,24,29–33]. Almost all studies (n = 19) used blood samples as influenza A (H1N1) diagnostic method [16,19–21,23–26,28–38]. Six studies (30%) used nasopharyngeal swabs as additional diagnostic method [20,21,24,28,36,37]. The kind of diagnostic method, blood sample or nasopharyngeal swab, has no impact on the prevalence rate of influenza A (H1N1) among HCP or controls/comparisons (data not shown). The sample size ranged from 66 to 15,018 participants. Prevalence rates for 2009 influenza A (H1N1) infection among HCP varied from 1.7% to 27.1%. Eight out of twenty studies (40%) used population-based controls (e.g. general population and non-HCP), eleven (55%) used hospital-based comparisons (e.g. nurses, technicians, students, auxiliary, support and administrative staff) and one study (5%) used reference data from the general population in their study. Prevalence rates among controls/comparisons ranged between 1.0% and 30.0%. Nine high quality [16,26,29–31,33–35,37] and two moderate quality [38,39] studies showed higher H1N1 prevalence rates for HCP compared to controls/comparisons. Based on the eight quality criteria, fourteen studies (70%) were of high quality (≥ 10 points) [16,21,23,24,26,29–37,40,41] and six studies (30%) of moderate quality (≥ 5 points) [19,20,25,28,38,39]. The most frequent reasons for moderate quality were weaknesses relating to the response rate, description of non-responders, control group and confirmatory test.

**Table 2:** Study characteristics of included studies reporting influenza A (H1N1) infection among HCP (n = 26).

Reference	Country	Setting	Study population	Diagnostic test/ time of sera collection	Sample size	HCP: n (n+)	Preva-lence %	Controls/ comparisons^a^	Controls/ comparesons: n (n+)	Preva-lence %	Quality score ^b^
Cross-sectional studies											
Aguilar-Madrid 2015 [33]	Mexico	Medical centres, hospitals, clinics	Physicians, nurses, laboratory staff, administrative staff, technicians, therapists, other HCP	HAI, MN(n/a)	1,143	1,090 (296)	27.1	P (community adults)	53 (11)	20.7	High (13)
Alagappan 2013 [34]	USA	Tertiary care hospitals -emergency & acute care departments, influenza units	Physicians, nurses	HAI, M (28.10.-16.12.09)	340	193 (41)	21.2	P (non-HCP adults)	147 (24)	16.3	High (11)^1-5, 8–10, 12–14^
Bandaranayake 2010 [29,40]	New Zealand	General practices, hospitals	Medical staff, nursing staff, allied health/ support staff	HAI (Nov.09-Mar.10)	1,053	532 (142)	26.6	P (children & adults)	521 (62)	11.9	High (13)^1-6, 8–14^
Costa 2012 [21]	Portugal	Tertiary care teaching hospital	Physicians, nurses, auxiliary staff, administrative staff	RT-PCR (n/a)	5,592	4,648 (91)	1.9^c^	HC (administra-tive/other staff)	944 (6)	0.6c	High (10)^1-5, 8, 10, 12–14^
Hudson 2013 [35]	New Zealand	General practices	General practitioners, practice nurses, receptionists	HAI (Dec.09-Feb.10)	1,005	681 (153)	22.4	HC (receptionists)	324 (71)	21.9	High (11)^1-6, 8, 10, 12–14^
Kumar 2011 [28]	USA	Hospitals -emergency departments	Physicians, nurses, therapists, paramedical staff	HAI, RT-PCR (n/a)	108	108 (20)	18.5	PR (community adults)	262 (7)	2.6	Moderate (7)^1-4, 10, 12, 14^
Nukui 2012 [25]	Japan	Acute care hospital	Physicians, nurses, co-medical staff	HAI (14.09.-04.10.09)	461	438 (27)	6.1	HC (co-medical staff)d	23 (n/a)	n/a	Moderate (8)^1-4, 8, 10, 13–14^
Olalla 2012 [38]	Spain	Hospital	Physicians, nurses, nursing assistants, orderlies/ administrative staff	HAI (25.08.-16.09.09)	239	225 (57)	25.3	HC (administrative staff)	14 (3)	21.4	Moderate (9)^1-3, 5, 8, 10, 12–14^
Smit 2012 [20]	The Netherlands	Hospital	Physicians, nurses, doctors assistants, technicians, other HCP	HAI, RT-PCR (17.08.09–08.01.10)	66	46 (1)	2.1^c^	HC (low risk group)	20 (0)	-^c^	Moderate (6) ^1, 3, 8, 10, 12, 14^
Smith 2011 [26]	Scotland	Acute care hospital	Physicians, nurses, midwives, allied health professionals, laboratory/ administrative staff, students, other HCP	MN (n/a)	n/a	493 (51)	10.3	HC (non-frontline HCP)	n/a (n/a)	9,1	High (10)^1-6, 8, 10, 12, 14^
Tandale 2010 [16]	India	Hospitals, general practices	Physicians, general practitioners, nurses, support staff	HAI, RT-PCR (n/a)	3,183	663 (72)	10.8	P (families)	2,520 (151)	5.9	High (11)^1-5, 8–12, 14^
Toyokawa 2011 [31]	Japan	Hospitals -emergency departments, influenza units	Physicians, nurses, technicians, pharmacists	HAI (18.06.-10.07.09)	268	209 (11)	5.2	HC (technicians/ pharmacists)	59 (3)	5.0	High (11)^1-6, 8, 10–12, 14^
Yeom 2011 [39]	Korea	Hospitals	Physicians, nurses, nursing assistants, therapists, technicians, other HCP	RT-PCR (n/a)	15,018	10,318 (281)	2.7	HC (group IV)	4,700 (47)	1.0	Moderate (6)^1-2, 8, 10, 12, 14^
Zhou 2011 [32]	China	(Non-) acute care hospitals	Physicians, nurses, support staff	HAI, MN (n/a)	555	411 (40)	9.7	HC (non-clinical staff)	144 (24)	16.6	High (13)^1-8, 10–14^
Cohort studies											
Chen 2010 [30,41]	Singapore	Acute care hospital, nursing homes	Acute care hospital staff, staff of long-term care facilities	HAI, RT-PCR (22.06.-15.10.09)	1,396	558 (37)	6.6	P (community-dwelling adults)	838 (22)	2.6	High (11)^1-5, 9–14^
Jaeger 2011 [19]	USA	Tertiary care hospital, outpatient clinic—emergency departments	Clinical practitioners, allied health/support staff	HAI, MN, RT-PCR (n/a)	63	57 (9)	15.7c	HC (support staff)	6 (0)	-^c^	Moderate (6)^1-2, 8, 10, 12, 14^
Kuster 2013 [36]	Canada	Acute care hospital	Physicians, nurses, therapists	HAI, RT-PCR (29.05.-27.09.09 & Apr.-May 10)	732	563 (10)	1.7	P (office staff)	169 (6)	3.5	High (10)^1-3, 5, 8–10, 12–14^
Lee 2010 [23]	Singapore	Primary care medical centres	HCP	HAI (22.06.-01.07.09 & 20.08.-03.09.09 & 29.09.-09.10.09)	1,015	108 (12)	11.1	P (soldiers)	907 (273)	30.0	High (14)^1-14^
Marshall 2011 [37]	Australia	Tertiary care hospitals	Physicians, nurses, therapists, other HCP	HAI, RT-PCR (n/a)	446	231 (46)	19.9	P (librarians, IT/administrative staff)	215 (33)	15.3	^High (10)1-3, 5, 8–10, 12–14^
Yen 2012 [24]	Taiwan	Tertiary medical centre	Physicians, nurses, technicians	HAI, RIDT, RT-PCR, VI (Aug.09, Oct.09 & Mar.10)	282	150 (18)	12.0^c^	HC (low risk group)^d^	n/a (n/a)	n/a	High (12) ^1–5, 7–8, 10–14^
Surveillance studies											
Balkhy 2010^e^ [22]	Saudi Arabia	Tertiary care medical centre	Physicians, nurses, nursing assistants, therapists, technicians, other HCP	RT-PCR (n/a)	9,780	6,415 (382)	5.9^c^	P (administra-tive/support staff)	3,365 (144)	4.2^c^	Moderate (9) ^1–5, 8–10, 12^
Chan 2010^f^ [42]	Taiwan	Medical centre	Physicians, nurses, laboratory/ administrative staff, students	HAI (23.10.-20.11.09)	539	295 (59)	20.0	P (people who came for physical check-up)	244 (7)	2.8	Moderate (9)^1-3, 5, 8–10, 12,14^
Seto 2011^e^ [43]	China	Hospitals, clinics	Nurses, healthcare assistants, medical staff, allied health professionals	RT-PCR, VC (n/a)	59,270	40,511 (1,039)	2.5	HC (non-clinical staff)	18,759 (119)	0.6	Moderate (9)^1-5, 8, 10, 12, 14^
Content analyses											
Bhadelia 2013 [27]	USA	Tertiary care medical centre	HCP	ELISA, IFA, RT-PCR, VC (n/a)	393	352 (141)	40.0	HC (negative influenza A cases) ^d^	n/a (n/a)	n/a	Moderate (8) ^1–5, 10, 12, 14^
Santos 2010 [44]	USA	Hospital	Physicians, nurses, technicians, administrative staff, other HCP	RIDT, RT-PCR (n/a)	6,093	2,806 (84)	2.9	HC (administra-tive/support staff)	3,287 (39)	1.1	Moderate (6) ^1–2, 8, 10, 12, 14^
Case-case-control study											
Lobo 2013 [6]	Brazil	Tertiary care hospital	Physicians, nurses, nurse technicians, administrative staff, students	RT-PCR (n/a)	282	180 (52)	28.8	H (HCP without respiratory symptoms)^d^	102 (n/a)	n/a	High (11) ^1–6, 8–10, 12–13^

Abbreviations: ELISA: enzyme-linked immunosorbent assay, H: hospital-based controls, HAI: haemagglutination inhibition test, HC: hospital-based comparisons, HCP: healthcare personnel, IFA: immunofluorescent antibody test, MN: microneutralisation test, n/a: not applicable, P: population-based controls, PR: reference data on population-based controls, RIDT: rapid influenza diagnostic test, RT-PCR: reverse-transcription polymerase chain reaction, USA: United States of America, VC: viral culture, VI: viral isolation.

^a^comparisons were selected that are most similar to population-based controls (e.g. administrative/support staff, receptionists),

^b^details for criteria see Table 1,

^c^in this case: incidence %,

^d^data was not analysed for subgroups separately,

^e^HCP was tested with presented influenza-like illness (ILI) symptoms,

^f^presence of ILI symptoms was not reported.

Similarly to the observational studies, three out of the six other studies were carried out in Asia (Saudi Arabia, Taiwan and China). Three studies were based in North and South America (United States of America and Brazil). Half of the studies (n = 3) selected tertiary care hospitals/medical centres as the study location. The remaining studies (n = 3) were conducted in general hospitals/medical centres. All six studies performed laboratory tests, with RT-PCR being the most-used testing method (n = 5). For all studies the utilisation of a confirmatory test is unknown [6,22,27,42–44]. Four out of six studies (67%) used blood samples as diagnostic method [27,42–44]. Two studies (33%) used nasopharyngeal swabs [6,22]. The sample size varied from 282 to 59,270 study subjects. Prevalence rates for HCP ranged between 2.5% and 40.0%. Three studies (50%) involved control groups in their study, two studies population-based controls (e.g. administrative staff) and one study a hospital-based control (asymptomatic HCP, e.g. nurses and nurse technicians). The remaining studies (n = 3) used hospital-based comparisons (e.g. healthcare assistants and administrative staff). Prevalence rates for controls/comparisons varied from 0.6% to 2.8%. Three moderate quality studies [42–44] found higher H1N1 prevalence rates for HCP compared to controls/comparisons. Five out of six studies (83%) were of moderate [22,27,42–44] and one study (17%) of high quality [6].

Several studies examined the job-related effect estimate (Odds Ratio, Relative Risk) of 2009 pandemic influenza A (H1N1) infection among HCP (**Table 3**). The results show that risk estimates vary from study to study. Three high quality studies [6,21,41] and one moderate quality study [25] report a significantly higher risk for HCP compared to controls/comparisons. One study found that the odds were six times higher for physicians in comparison with other professions (e.g. technicians; OR = 6.03, CI = 2.11 to 17.82) [6]. Nukui and colleagues showed similar results. In this study physicians and nurses ran a five times higher odds of influenza A (H1N1) infection than co-medical staff (OR = 5.25, CI = 1.21 to 22.7) [25]. Another study reported a significantly increased odds of influenza A (H1N1) infection for nurses compared to administrative or other staff (OR = 2.7, CI = 1.11 to 6.37) [21]. Chen and colleagues found consistent results for nurses in comparison with allied health staff (OR = 6.1, CI = 1.4 to 26.0) [41]. In contrast to this, one high quality study found a significantly lower risk for HCP compared to other personnel (e.g. soldiers; RR = 0.26, CI = 0.07 to 0.46) [23]. Six high quality studies [32–37] and one moderate quality study [43] showed no significantly higher or lower risk of influenza A (H1N1) infection for HCP in comparison with non-HCP.

**Table 3:** Selected studies analysing the job-related effect estimate (OR, RR) of influenza A (H1N1) infection among HCP (n = 13).

Reference	Occupation/staff	n (all)^a^	%	n (+)	%	Risk estimate with 95% CI
Aguilar-Madrid [33]	Staff with <5 contacts to patients with suspected 2009 pandemic influenza A (H1N1) infection	1,063	49.4	262	24.6	1 -
	Physicians	466	21.6	144	30.9	OR = 1.31 (0.93–1.84)
	Nurses. medical assistants	624	29.0	152	24.3	OR = 0.95 (0.68–1.32)
Alagappan 2013 [34]	Non-HCP	147	43.2	24	16.3	1 -
	HCP	193	56.8	41	21.2	COR = 1.35 (0.77–2.36)
Chen 2010 [41]	Allied health staff	116	21.8	2	1.7	1 -
	Physicians	21	4.0	1	4.8	COR = 2.9 (0.2–32.9); AOR = 3.8 (0.5–28.7)
	Nurses	290	54.6	28	9.7	COR = 6.1 (1.4–26.0); AOR = 4.5 (1.0–19.6)
	Auxiliary/support staff	69	13.0	2	2.9	COR = 1.7 (0.2–12.4); AOR = 1.5 (0.2–11.1)
	Administrative staff	35	6.6	2	5.7	COR = 3.5 (0.5–25.5); AOR = 3.6 (0.3–42.8)
Costa 2012 [21]	Administrative or other staff	944	16.9	6	0.6	1 -
	Physicians	1,393	24.9	19	1.4	OR = 1.8 (0.71–4.62)
	Nurses	1,982	35.4	56	2.8	OR = 2.7 (1.11–6.37); AOR = 3.8 (1.2–6.8)
	Auxiliary staff	1.273	22.8	16	1.3	OR = 1.4 (0.55–3.65)
Hudson 2013 [35]	Receptionists	324	32.2	71	21.9	1 -
	GPs	294	29.3	63	21.4	OR = 1.0 (0.7–1.4)
	Nurses	387	38.5	90	23.3	OR = 1.1 (0.8–1.5)
Kuster 2013 [36]	Non-HCP	169	23.1	6	3.6	1 -
	HCP	563	76.9	10	1.8	AOR = 0.49b (0.19–1.27); AOR = 0.47c (0.17–1.32)
Lee 2010 [23]	Other personnel	437	43.0	n/a	n/a	1 -
	Essential personnel	470	46.3	n/a	n/a	RR = 0.39 (0.26–0.54)
	HCP	108	10.7	12	11.0	RR = 0.26 (0.07–0.46)
Lobo 2013 [6]	Other professions	131	85.0	36	27.5	1 -
	Physicians	23	15.0	16	69.6	OR = 6.03^b^ (2.11–17.82); OR = 8.58c (2.52–29.27)
Marshall 2011 [37]	Non-clinical staff	215	48.2	33	15.3	1 -
	Clinical staff	231	51.8	46	19.9	OR = 1.37 (0.84–2.22)
Nukui 2012 [25]	Co-medical staff	23	5.0	n/a	n/a	1 -
	Physicians/nurses	438	95.0	27	6.1	OR = 5.25 (1.21–22.7)
	Other medical staff	83	19.0	16	19.3	1 -
	Internal medicine/ emergency/paediatrics staff	355	81.0	130	36.6	COR = 2.42 (1.35–4.35); AOR = 1.98 (1.07–3.65)
Olalla 2012 [38]	Nurses	73	30.5	8	10.9	1 -
	Physicians	65	27.2	20	30.8	OR = 4.08 (1.48–11.22)
	Auxiliary nursing staff	63	26.4	19	30.2	OR = 2.33 (0.48–11.35)
	Orderlies	24	10.0	10	41.7	OR = 5.01 (1.79–14.01)
	Administrative staff	14	5.9	3	21.4	OR = 4.83 (1.42–16.46)
Seto 2011 [43]	Non-clinical staff	18,769	31.7	119	0.6	1 -
	Clinical staff	40,511	68.3	1,039	2.5	RR = 0.98 (0.78–1.20)
Zhou 2011 [32]	Internal medicine staff	83	13.8	8	9.6	1 -
	Surgery staff	54	9.0	8	14.8	COR = 1.58 (0.56–4.52); AOR = 1.57 (0.54–4.57)
	Emergency room staff	9	1.5	3	33.3	COR = 4.53 (0.94–21.89); AOR = 4.56 (0.91–22.87)
	Paediatrics staff	38	6.3	4	10.5	COR = 1.06 (0.30–3.75); AOR = 1.07 (0.30–3.87)
	Other clinical dep. staff	255	42.5	30	11.7	COR = 1.24 (0.54–2.84); AOR = 1.33 (0.57–3.09)
	Non-clinical staff	147^d^	24.5	21	14.2	COR = 1.46 (0.61–3.49); AOR = 2.07 (0.84–5.12)

Abbreviations: AOR: adjusted odds ratio, CI: confidence interval, COR: crude odds ratio, dep: department, GPs: general practitioners, HCP: healthcare personnel, n/a: not applicable, OR: odds ratio, RR: relative risk,

^a^ data can vary from numbers shown in Table 2 as the database can be different in several analyses of a study,

^b^ univariate analysis,

^c^ multivariate analysis,

^d^ unknown: 13 (2.2%).

In addition, several studies [21,29,34–36] examined non-occupational risk factors such as gender, age, children at home, vaccination history and influenza-like illness symptoms for HCP to contract influenza A (H1N1) infection. Moreover, the personal protective equipment (PPE) use among HCP is a frequently investigated topic in infectious diseases research. Many studies analysed the efficacy of PPE like surgical masks, gowns, gloves, goggles, N95 respirators and face shields as well as of hand hygiene [5,19,24,25,37,39,43]. The studies mainly concluded that an adequate PPE use and hand hygiene decrease the risk of influenza A (H1N1) infection among HCP (not shown).

### Meta-Analysis

**Fig 2:**
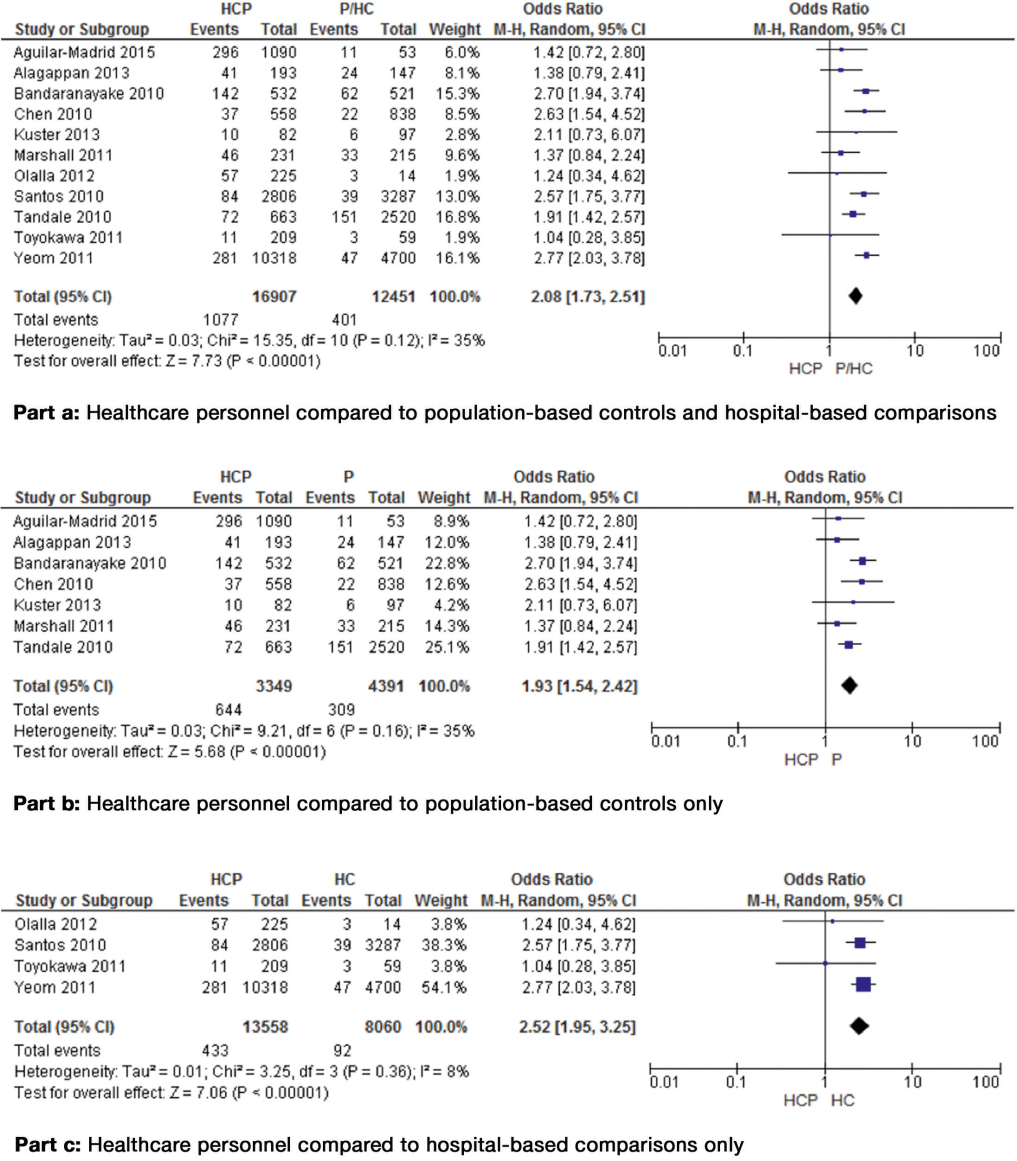
**Forest plots showing the risk of influenza A (H1N1) infection for HCP compared to population-based controls/hospital-based comparisons (part a) and controls/comparisons only (part b and c).** Abbreviations: CI: confidence interval, df: degrees of freedom, HC: hospital-based comparisons, HCP: healthcare personnel, M-H: Mantel-Haenszel, P: population-based controls.

Fifteen studies (58%) were included in the meta-analysis (**S1 Fig**), yielding an OR of 1.94 (95% CI = 1.40 to 2.70). Because of high evidence of heterogeneity among the studies (χ² = 115.97, p < 0.00001, I² = 88%) four studies [32,35,42,43] were identified as sources of methodological variability and were excluded from the pooled analysis. The meta-analysis comprised 29,358 subjects (HCP and P/HC) from eleven studies (8 high quality and 3 moderate quality studies). Of these, 1,478 had influenza A (H1N1)-positive test results (**Fig 2**, part a). The study population in our meta-analysis included for example physicians, nurses, therapists, laboratory staff and general practitioners. The control groups were all population-based, the comparison groups all hospital-based. In order to adequately address the diversity and heterogeneity of the limited number of studies included in the meta-analysis, the pooled effect estimate was determined using the random effect model [17]. Approximately half of the included subjects (51.2%) were vaccinated against influenza A (H1N1). In two studies the vaccination status was unknown [16,37]. The pooled prevalence rate was 5.0% (for HCP only: 6.3%). In addition, the pooled analysis showed that the odds for an influenza A (H1N1) infection were significantly higher for HCP compared to all controls/comparisons (OR = 2.08, 95% CI = 1.73 to 2.51). The χ² test revealed no evidence of heterogeneity among the studies (χ² = 15.35, p = 0.12), the I² value was 35% (**Fig 2**, part a). Pooling the studies with high methodological quality only (n = 7) showed also a significantly increased OR of 1.75 (95% CI = 1.44 to 2.13) for an influenza A (H1N1) infection among HCP in comparison with controls/comparisons, with no evidence of heterogeneity (χ² = 5.28, p = 0.51, I^2^ = 0%; plot not shown). The pooled analysis comprised 6,955 subjects, with 763 H1N1-infected subjects. The pooled prevalence rate increased to 10.9% (for HCP only: 16.9%).

After stratification by control group, the pooled analysis with population-based controls only showed a significantly almost two times higher odds for an influenza A (H1N1) infection for HCP (OR = 1.93, 95% CI = 1.54 to 2.42; **Fig 2**, part b). The pooled analysis was performed including seven studies, one study [42] was excluded after testing for heterogeneity. The analysis included 7,740 subjects. Of these 953 had influenza A (H1N1)-positive test results. The pooled prevalence rate was 12.3% (for HCP only: 19.2%). The χ² test showed no evidence of heterogeneity among the studies (χ² = 9.21, p = 0.16), the I² value was 35%. All included studies were of high methodological quality.

The pooled analysis with hospital-based comparisons only comprised 21,618 subjects, with 525 being H1N1-infected (**Fig 2**, part c). The pooled prevalence rate was 2.4% (for HCP only: 3.2%). The odds ratio was significantly increased by 2.52 (95% CI = 1.95 to 3.25) for an influenza A (H1N1) infection among HCP compared to comparisons, with no evidence of heterogeneity (χ² = 3.25, p = 0.36, I² = 8%). The analysis was performed with four studies, three studies [32,35,43] were excluded after testing for heterogeneity. This analysis comprised only one high methodological quality study.

#### Heterogeneity and sensitivity analysis

Heterogeneity was present when pooling all studies with population-based controls/hospital-based comparisons (**S1 Fig**). To identify sources of heterogeneity a comprehensive investigation of the data was performed. A sensitivity analysis was conducted to explore the influence of each study on the overall results step by step to determine the robustness of the pooled effect estimates. The odds ratios calculated in the sensitivity analysis ranged from 1.98 (1.63 to 2.41) to 2.20 (1.85 to 2.63). They were all statistically significant. The I² value ranged from 23% to 41%. The pooled estimates and I² of the studies were stable within the analysis after exclusion of four studies that showed high effects on the pooled OR and I² (plots not shown). After stratification by control/comparison group the sensitivity analysis revealed that studies with large sample sizes have no significant influence on the pooled effect estimate.

In this meta-analysis, the included studies used population-based controls as control group or hospital-based comparisons as comparison group, HAI as influenza A (H1N1) detection method (with two exceptions) [39,44] and serum samples as diagnostic method (unknown in one case) [39], with three studies using nasopharyngeal swabs as additional diagnostic method [36,37,44].

#### Publication bias

Visual examination of the funnel plot (OR x SE (log [OR])) to assess publication bias revealed no significant asymmetry (plot not shown). However, this could be incidental because of the small number of studies included in the meta-analysis. Therefore we provided an Egger’s linear regression. The linear regression did not show significant funnel plot asymmetry either (intercept 0.11, 90% CI = 0.28 to 0.50, p = 0.61).

## Discussion

To our knowledge the present paper is the first systematic review and meta-analysis to examine the occupational risk of 2009 pandemic influenza A (H1N1) infection among healthcare personnel. In this literature review prevalence rates for 2009 influenza A (H1N1) infection among HCP varied from 1.7% to 27.1% and among controls/comparisons from 1.0% to 30% (see Table 2). Eleven out of 20 observational studies (55%; 9 out of 14 high quality and 2 out of 6 moderate quality studies) showed higher H1N1 prevalence rates for HCP compared to controls/comparisons [16,26,29–31,33–35,37–39]. Aguilar-Madrid and colleagues found a significantly increased odds for an influenza A (H1N1) infection among HCP with >5 contacts to probably infected patients compared to HCP with <5 contacts (OR = 1.47, 95% CI = 1.11 to 1.94) [33]. It is important, however, to mention that the exposure of some hospital-based comparisons could not be clarified. In our meta-analysis the pooled prevalence rate for HCP alone was 6.3% and for all included subjects 5.0%.

The pooled analysis further showed a significantly increased OR of 2.08 (95% CI = 1.73 to 2.51) for an influenza A (H1N1) infection among HCP compared to controls/comparisons. The stratified analysis with population-based controls (OR = 1.93, 95% CI = 1.54 to 2.42) and hospital-based comparisons (OR = 2.52, 95% CI = 1.95 to 3.25) confirmed the significantly increased odds of influenza A (H1N1) infection for HCP. Stratified by occupation several high quality studies [6,21,41] and one moderate quality study [25] showed that physicians and nurses are particularly at risk for influenza A (H1N1) infection (e.g. OR = 5.25, 95% CI = 1.21 to 22.7; see Table 3). Bernard and colleagues also stated that HCP are most likely to be at greater risk of contracting influenza A (H1N1) than the general population due to their constant close contact with infected patients [7]. However, compared with other professions, hospital staff and general practitioners had a significantly lower risk of infection than school staff and schoolchildren [16]. The presence of children in households is a well-known risk factor for an influenza A (H1N1) infection [28,30,34] of its own. School staff are also particularly exposed to H1N1 microbes due to their daily work with children, but do not use PPE as HCP do. Similar results are shown in a study by Lee and colleagues [23]. This high quality study found that the risk of infection among HCP was significantly lower than that of staff working as soldiers in military units (RR = 0.26, 95% CI = 0.07 to 0.46). The authors assumed that HCP were at lower risk of infection because of their regularly use of PPE during working hours. School staff and soldiers alike do not use such equipment at work [23]. Several high [24,37] and moderate [19,25,43] quality studies also proved that an adequate PPE use like surgical masks, gowns, gloves and goggles reduces the risk of pandemic influenza A (H1N1) infection.

Several included studies also analysed other risk factors likely to lead to an increased risk of H1N1 infection among HCP. Apart from the occupational risk, non-occupational risk factors such as sex, age, vaccination history, influenza-like illness symptoms and other household members were examined. Alagappan and colleagues and Kuster and colleagues found that female sex, younger age, previous influenza-like illness and children under 18 years of age at home are risk factors for contracting influenza A (H1N1) infection among HCP [34,36]. Three other studies showed similar results [21,29,35]. Moreover, study subjects who received a seasonal influenza vaccine were twice as likely to be infected with influenza A (H1N1) virus than unvaccinated subjects [21,35]. The authors of the high quality studies conclude that seasonal influenza vaccination had no protective effect against the 2009 pandemic [21,35]. Hudson and colleagues state that this observation may be due to cross-reactivity of antibodies induced by seasonal vaccination to influenza A (H1N1) [35]. Because of the missing protective effect of seasonal influenza vaccination especially during the first period of pandemics it is important that HCP adequately use PPE as prevention strategy against an infection. These findings also emphasize that the risk of H1N1 infection among HCP depends on several factors. However, the results of this study show that the occupation significantly contributes to the risk of infection. Some studies [45–47] confirmed the occupational risk among HCP for other infections than influenza A (H1N1). The authors found an increased risk of infection among HCP for the severe acute respiratory syndrome (SARS), avian influenza (H5N1) infection, human immunodeficiency virus (HIV) infection, hepatitis and tuberculosis. Therefore, it can be stated that healthcare is a setting in which employees are particularly vulnerable for acquiring infectious diseases.

### Strengths and limitations

This literature review included 26 out of 93 studies (28%) that were identified after the removal of duplicates. This is a small number of studies. However, the included sources clearly met the eligibility criteria and showed relevant data for the review. Nine studies did not fulfil the criteria and were excluded from full-text screening [5,48–55]. Only in a few cases, data is missing (see Tables 2 and 3). Moreover, all studies were of moderate or high quality (6 to 14 points). The quality assessment revealed some weaknesses in methodology. Twenty-two studies (85%) did not include a description of non-responders, 18 studies (69%) did not achieve an adequate response rate (≥ 60%) and did not perform a confirmatory laboratory test to detect influenza A (H1N1) virus among the study subjects. Another fifteen studies (58%) did not include a control group in their investigation.

Fifteen studies could be included in the meta-analysis. Eleven studies were excluded before conducting the analysis. Reasons for exclusion have been discussed above. In addition, the studies used different methods to perform investigations. Study populations, case definitions and cut-off values for seropositivity of influenza A (H1N1) vary from study to study. According to the literature the cut-off value for seropositivity is for HAI ≥ 1:40 [16]. Only one study defined a test result already as seropositive for influenza A (H1N1) if the HAI antibody titre was ≥1:20 [34]. Seven studies used the current cut-off value of ≥ 1:40 [16,29–31,33,37,38]. In one study the case definition is not clear as the authors used different cut-off values for seropositivity depending on the vaccination status [36]. Two studies used RT-PCR as the only diagnostic method [39,44]. The type of diagnostic test, HAI or RT-PCR, has no influence on the results of our meta-analysis. Both tests are comparable in terms of measurement and validity.

However, the strength of our meta-analysis is that the heterogeneity between the studies is low as the pooled estimates and I² values of the studies were stable within the analysis after identification and exclusion of a few studies as sources of heterogeneity. All included studies used population-based controls as control group, hospital-based comparisons as comparison group, HAI as influenza A (H1N1) detection method (with two exceptions; RT-PCR only) [39,44] and serum samples as diagnostic method (with one unknown) [39]. Three studies [36,37,44] used nasopharyngeal swabs as additional testing method. Almost all studies were of high quality (8 out of 11) and had a good study design (cross-sectional study or cohort study). The forest plot also indicates that the confidence intervals belonging to the odds ratios overlap widely. It can be assumed that the degree of inconsistency between the studies is low [56]. Finally, the stratification by control and comparison group confirmed the higher odds of influenza A (H1N1) infection among HCP compared to controls/comparisons.

## Conclusions

The 2009 pandemic influenza A (H1N1) virus infections played an important role in healthcare settings. This systematic review corroborates the assumption that HCP were particularly at risk of influenza A (H1N1) infection during the 2009 pandemic. The pooled analysis showed a statistically significant increase in the odds of influenza A (H1N1) infection among HCP compared to controls/comparisons. Our findings can help to enhance infection control especially during the first period of pandemics. Due to close contact with infected patients in healthcare settings and decreased protective effect of vaccinations, healthcare facilities should intensify their focus on strategies to prevent an infection among HCP. Well-known strategies such as PPE and hand hygiene are suitable, but HCP should be properly educated in these infection control practices.

## Supporting Information

S1 Appendix Detailed search strategies of all included databases. (PDF)

S1 Fig Forest plot showing all included studies of the meta-analysis (before sensitivity analysis). (TIF)

S1 File MOOSE checklist. (PDF)

S1 Table Prisma checklist pdf (PDF)

## Abbreviations

AOR: adjusted odds ratio
CI: confidence interval
COR: crude odds ratio
dep: department
df: degrees of freedom
ELISA: enzyme-linked immunosorbent assay
GPs: general practitioners
H: hospital-based control
HAI: haemagglutination inhibition test
HC: hospital-based comparison
HCP: healthcare personnel
IFA: immunofluorescent antibody test
M-H: Mantel-Haenszel
MN: microneutralisation test
MOOSE: meta-analysis of observational studies in epidemiology
n/a: not applicable
non-HCP: non-healthcare personnel
OR: odds ratio
P: population-based control
PPE: personal protective equipment
PR: reference data on population-based controls
PRISMA: preferred reporting items for systematic review and meta-analysis
RIDT: rapid influenza diagnostic test
RR: relative risk
RT-PCR: reverse-transcription polymerase chain reaction
SE: standard error
USA: United States of America
VC: viral culture
VI: viral isolation
