# Supplementary material for: The Occupational Risk of Influenza A (H1N1) Infection among Healthcare Personnel during the 2009 Pandemic: A Systematic Review and Meta-Analysis of Observational Studies
Source: PLoS One. 2016 Aug 31;11(8):e0162061. doi: 10.1371/journal.pone.0162061 (PMC5006982; doi:10.1371/journal.pone.0162061)
Supplement: S1 Appendix — (PDF) [file pone.0162061.s001.pdf]

S1_Appendix

1. Search strategy: MEDLINE (via OVID)

1 Influenza A Virus, H1N1 Subtype/ (12052)
2 Pandemics/ (3615)
3 Health Personnel/ (24737)
4 Occupational Exposure/ (44206)
5 1 and 2 and 3 and 4 (6)
6 limit 5 to yr="2009 -Current" (6)

2. Search strategy: PubMed

(((((influenza a virus, h1n1 subtype) AND pandemic*) AND health personnel) AND occupational exposure) Filters: Publication date from 2009/03/01 to 2015/06/22 = 19

3. Search strategy: CINAHL (via OVID)

Influenza virus a, subtype h1n1 AND pandemic AND health personnel AND occupational exposure, ab 2009 = 1

4. Search strategy: EMBASE (via OVID)

1 Influenza virus A H1N1/ (10515)
2 pandemic influenza/ (3646)
3 health care personnel/ (104866)
4 occupational exposure/ (68045)
5 1 and 2 and 3 and 4 (4)
6 limit 5 to yr="2009 -Current" (4)

5. Search strategy: PsycINFO

1 influenza a H1N1.mp. [mp=ti, ab, sh, id, hw, tc, ot, tm, ax, kp, fw, cw, ia] (111)
2 pandemic*.mp. [mp=ti, ab, sh, id, hw, tc, ot, tm, ax, kp, fw, cw, ia] (1457)
3 health personnel.mp. [mp=ti, ab, sh, id, hw, tc, ot, tm, ax, kp, fw, cw, ia] (38514)
4 occupational exposure.mp. [mp=ti, ab, sh, id, hw, tc, ot, tm, ax, kp, fw, cw, ia] (1333)
5 1 and 2 and 3 and 4 (0)

6. Search strategy: Google Scholar

Search terms German: Influenza a h1n1, Pandemie, Beschäftigte im Gesundheitswesen, berufliche Exposition Filter: Publikationen ab 2009 bis heute = 2
Search terms English: influenza a h1n1, pandemic, health personnel, health care workers, occupational exposure, occupational risk Filters: Publication date from 2009 to current = 42
