# Supplementary figures and images for: The Occupational Risk of Influenza A (H1N1) Infection among Healthcare Personnel during the 2009 Pandemic: A Systematic Review and Meta-Analysis of Observational Studies
Source: PLoS One. 2016 Aug 31;11(8):e0162061. doi: 10.1371/journal.pone.0162061 (PMC5006982; doi:10.1371/journal.pone.0162061)

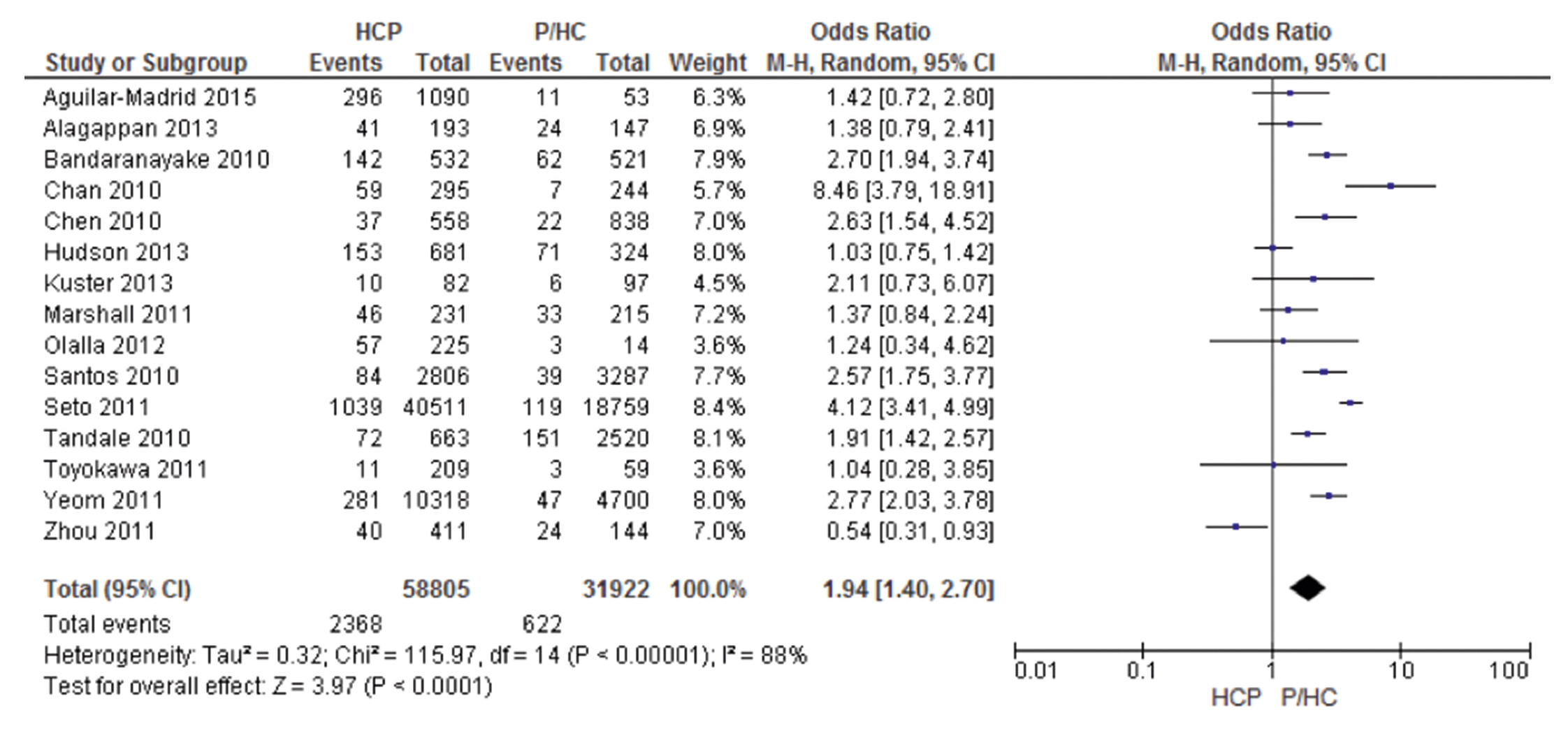

Supplement: S1 Fig — (TIF) [file pone.0162061.s002.tif]
