# Supplementary material for: The Occupational Risk of Influenza A (H1N1) Infection among Healthcare Personnel during the 2009 Pandemic: A Systematic Review and Meta-Analysis of Observational Studies
Source: PLoS One. 2016 Aug 31;11(8):e0162061. doi: 10.1371/journal.pone.0162061 (PMC5006982; doi:10.1371/journal.pone.0162061)
Supplement: S1 File — (PDF) [file pone.0162061.s003.pdf]

Meta-analysis of Observational Studies in Epidemiology

A Proposal for Reporting

Donna F. Stroup, PhD, MSc
Jesse A. Berlin, ScD
Sally C. Morton, PhD
Ingram Olkin, PhD
G. David Williamson, PhD
Drummond Rennie, MD
David Moher, MSc
Betsy J. Becker, PhD
Theresa Ann Sipe, PhD
Stephen B. Thacker, MD, MSc
for the Meta-analysis Of Observational Studies in Epidemiology (MOOSE) Group

Objective Because of the pressure for timely, informed decisions in public health and clinical practice and the explosion of information in the scientific literature, research results must be synthesized. Meta-analyses are increasingly used to address this problem, and they often evaluate observational studies. A workshop was held in Atlanta, Ga, in April 1997, to examine the reporting of meta-analyses of observational studies and to make recommendations to aid authors, reviewers, editors, and readers.

Participants Twenty-seven participants were selected by a steering committee, based on expertise in clinical practice, trials, statistics, epidemiology, social sciences, and biomedical editing. Deliberations of the workshop were open to other interested scientists. Funding for this activity was provided by the Centers for Disease Control and Prevention.

Evidence We conducted a systematic review of the published literature on the conduct and reporting of meta-analyses in observational studies using MEDLINE, Educational Research Information Center (ERIC), PsycLIT, and the Current Index to Statistics. We also examined reference lists of the 32 studies retrieved and contacted experts in the field. Participants were assigned to small-group discussions on the subjects of bias, searching and abstracting, heterogeneity, study categorization, and statistical methods.

Consensus Process From the material presented at the workshop, the authors developed a checklist summarizing recommendations for reporting meta-analyses of observational studies. The checklist and supporting evidence were circulated to all conference attendees and additional experts. All suggestions for revisions were addressed.

Conclusions The proposed checklist contains specifications for reporting of meta-analyses of observational studies in epidemiology, including background, search strategy, methods, results, discussion, and conclusion. Use of the checklist should improve the usefulness of meta-analyses for authors, reviewers, editors, readers, and decision makers. An evaluation plan is suggested and research areas are explored.

JAMA. 2000;283:2008-2012 www.jama.com

BECAUSE OF PRESSURE FOR TIMELY and informed decisions in public health and medicine and the explosion of information in the scientific literature, research results must be synthesized to answer urgent questions.[1-4] Principles of evidence-based methods to assess the effectiveness of health care interventions and set policy are cited increasingly.[5] Meta-analysis, a systematic approach to identifying, appraising, synthesizing, and (if appropriate) combining the results of relevant studies to arrive at conclusions about a body of research, has been applied with increasing frequency to randomized controlled trials (RCTs), which are considered to provide the strongest evidence regarding an intervention.[6,7]

However, in many situations randomized controlled designs are not feasible, and only data from observational studies are available.[8] Here, we define an observational study as an etiologic or effectiveness study using data from an existing database, a cross-sectional study, a case series, a case-control design, a design with historical controls, or a cohort design.[9] Observational designs may lack the experimental element of a random allocation to an intervention and rely on studies of association between changes or differences in 1 characteristic (eg, an exposure or intervention) and changes or differences in an outcome of

Author Affiliations: Centers for Disease Control and Prevention, Atlanta, Ga (Drs Stroup, Williamson, and Thacker); University of Pennsylvania School of Medicine, Philadelphia (Dr Berlin); RAND Corporation, Santa Monica (Dr Morton), University of California, San Francisco (Dr Rennie), Stanford University, Stanford (Dr Olkin), Calif; *JAMA*, Chicago, Ill (Dr Rennie); Thomas C. Chalmers Centre for Systematic Reviews, Children's Hospital of Eastern Ontario Research Institute, Ottawa (Mr Moher); Michigan State University, East Lansing (Dr Becker); and Georgia State University, Atlanta (Dr Sipe).
A complete list of members of the MOOSE Group appears at the end of this article.
Corresponding Author and Reprints: Donna F. Stroup, PhD, MSc, Centers for Disease Control and Prevention, 1600 Clifton Rd NE, Mail Stop C08, Atlanta, GA 30333 (e-mail: dfs2@cdc.gov).

 ©2000 American Medical Association. All rights reserved.

interest. These designs have long been used in the evaluation of educational programs[10] and exposures that might cause disease or injury.[11] Studies of risk factors generally cannot be randomized because they relate to inherent human characteristics or practices, and exposing subjects to harmful risk factors is unethical.[12] At times, clinical data may be summarized in order to design a randomized comparison.[13] Observational data may also be needed to assess the effectiveness of an intervention in a community as opposed to the special setting of a controlled trial.[14] Thus, a clear understanding of the advantages and limitations of statistical syntheses of observational data is needed.[15]

Although meta-analysis restricted to RCTs is usually preferred to meta-analyses of observational studies,[16-18] the number of published meta-analyses concerning observational studies in health has increased substantially during the past 4 decades (678 in 1955-1992, 525 in 1992-1995, and more than 400 in 1996 alone).[19]

While guidelines for meta-analyses have been proposed, many are written from the meta-analyst's (author's) rather than from the reviewer's, editor's, or reader's perspective[20] and restrict attention to reporting of meta-analyses of RCTs.[21,22] Meta-analyses of observational studies present particular challenges because of inherent biases and differences in study designs[23]; yet, they may provide a tool for helping to understand and quantify sources of variability in results across studies.[24]

We describe here the results of a workshop held in Atlanta, Ga, in April 1997, to examine concerns regarding the reporting of Meta-analysis Of Observational Studies in Epidemiology (MOOSE). This article summarizes deliberations of 27 participants (the MOOSE group) of evidence leading to recommendations regarding the reporting of meta-analyses. Meta-analysis of individual-level data from different studies, sometimes called "pooled analysis" or "meta-analysis of individual patient data,"[25,26] has unique challenges that we will not address here. We propose a checklist of items for reporting that builds on similar activities for RCTs[22] and is intended for use by authors, reviewers, editors, and readers of meta-analyses of observational studies.

METHODS

We conducted a systematic review of the published literature on the conduct and reporting of meta-analyses in observational studies. Databases searched included MEDLINE, Educational Resources Information Center, PsycLIT (http://www.wesleyan.edu/libr), and the Current Index to Statistics. In addition, we examined reference lists and contacted experts in the field. We used the 32 articles retrieved to generate the conference agenda and set topics of bias, searching and abstracting, heterogeneity, study categorization, and statistical methods. We invited experts in meta-analysis from the fields of clinical practice, trials, statistics, epidemiology, social sciences, and biomedical editing.

The workshop included an overview of the quality of reporting of meta-analyses in education and the social sciences. Plenary talks were given on the topics set by the conference agenda. For each of 2 sessions, workshop participants were assigned to 1 of 5 small discussion groups, organized around the topic areas. For each group, 1 of the authors served as facilitator, and a recorder summarized points of discussion for issues to be presented to all participants. Time was provided for the 2 recorders and 2 facilitators for each topic to meet and prepare plenary presentations given to the entire group. We proposed a checklist for meta-analyses of observational studies based on the deliberation of the independent groups. Finally, we circulated the checklist for comment to all conference attendees and representatives of several constituencies who would use the checklist.

RESULTS

The checklist resulting from workgroup deliberations is organized around recommendations for reporting background, search strategy, methods, results, discussion, and conclusions (TABLE).

Background

Reporting of the background should include the definition of the problem under study, statement of hypothesis, description of the study outcome(s) considered, type of exposure or intervention used, type of study design used, and complete description of the study population. When combining observational studies, heterogeneity of populations (eg, US vs international studies), design (eg, case-control vs cohort studies), and outcome (eg, different studies yielding different relative risks that cannot be accounted for by sampling variation) is expected.[8]

Search

Reporting of the search strategy should include qualifications of the searchers, specification of databases used, search strategy and index terms, use of any special features (eg, "explosion"), search software used, use of hand searching and contact with authors, use of materials in languages other than English, use of unpublished material, and exclusion criteria used. Published research shows that use of electronic databases may find only half of all relevant studies, and contacting authors may be useful,[27] although this result may not be true for all topic areas.[28]

For example, a meta-analysis of depression in elderly medical inpatients[29] used 2 databases for the search. In addition, bibliographies of retrieved papers were searched. However, the authors did not report their search strategy in enough detail to allow replication. An example of a thorough "reject log" can be found in the report of a meta-analysis of electrical and magnetic field exposure and leukemia.[30] Examples of a table characterizing studies included can be found in Franceschi et al[31] and Saag et al.[32] Complete specification of search strategy is not uniform; a review of 103 published meta-analyses in education showed that search procedures were described inadequately in the majority of the articles.[10]

©2000 American Medical Association. All rights reserved.

Methods

Items in this checklist section are concerned with the appropriateness of any quantitative summary of the data; degree to which coding of data from the articles was specified and objective; assessment of confounding, study quality, and heterogeneity; use of statistical methods; and display of results. Empirical evidence shows that reporting of procedures for classification and coding and quality assessment is often incomplete: fewer than half of the meta-analyses reported details of classifying and coding the primary study data, and only 22% assessed quality of the primary studies.[10]

We recognize that the use of quality scoring in meta-analyses of observational studies is controversial, as it is for RCTs,[16,33] because scores constructed in an ad hoc fashion may lack demonstrated validity, and results may not be associated with quality.[34] Nevertheless, some particular aspects of study quality have been shown to be associated with effect: eg, adequate concealment of allocation in randomized trials.[35] Thus, key components of design, rather than aggregate scores themselves, may be important. For example, in a study of blinding (masking) of readers participating in meta-analyses, masking essentially made no difference in the summary odds ratios across the 5 meta-analyses.[36] We recommend the reporting of quality scoring if it has been done and also recommend subgroup or sensitivity analysis rather than using quality scores as weights in the analysis.[37,38]

While some control over heterogeneity of design may be accomplished through the use of exclusion rules, we recommend using broad inclusion criteria for studies, and then performing analyses relating design features to outcome.[8] In cases when heterogeneity of outcomes is particularly problematic, a single summary measure may well be inappropriate.[39] Analyses that stratify by study feature or regression analysis with design features as predictors can be useful in assessing whether study outcomes indeed vary systematically with these features.[40]

Investigating heterogeneity was a key feature of a meta-analysis of observational studies of asbestos exposure and risk of gastrointestinal cancer.[41] The authors of the meta-analysis hypothesized that studies allowing for a latent period between the initiation of exposure and any increases in risk should show, on average, appropriately higher standardized mortality ratios than studies that ignored latency. In other words, the apparent effect of exposure would be attenuated by including the latent period in the calculation of time at risk (the "denominator"), since exposure-related deaths (the "numerator") would, by definition, not occur during that latent period (FIGURE).

In fact, the data suggested that studies allowing for latent periods found on average somewhat higher standardized mortality ratios than studies ignoring latency. This example shows that sources of bias and heterogeneity can be hypothesized prior to analysis and subsequently confirmed by the analysis.

Results

Recommendations for reporting of results include graphical summaries of study estimates and any combined estimate, a table listing descriptive information for each study, results of sensitivity testing and any subgroup analysis, and an indication of statistical uncertainty of findings.

Table. A Proposed Reporting Checklist for Authors, Editors, and Reviewers of Meta-analyses of Observational Studies

- Reporting of background should include
  - Problem definition
  - Hypothesis statement
  - Description of study outcome(s)
  - Type of exposure or intervention used
  - Type of study designs used
  - Study population
- Reporting of search strategy should include
  - Qualifications of searchers (eg, librarians and investigators)
  - Search strategy, including time period included in the synthesis and keywords
  - Effort to include all available studies, including contact with authors
  - Databases and registries searched
  - Search software used, name and version, including special features used (eg, explosion)
  - Use of hand searching (eg, reference lists of obtained articles)
  - List of citations located and those excluded, including justification
  - Method of addressing articles published in languages other than English
  - Method of handling abstracts and unpublished studies
  - Description of any contact with authors
- Reporting of methods should include
  - Description of relevance or appropriateness of studies assembled for assessing the hypothesis to be tested
  - Rationale for the selection and coding of data (eg, sound clinical principles or convenience)
  - Documentation of how data were classified and coded (eg, multiple raters, blinding, and interrater reliability)
  - Assessment of confounding (eg, comparability of cases and controls in studies where appropriate)
  - Assessment of study quality, including blinding of quality assessors; stratification or regression on possible predictors of study results
  - Assessment of heterogeneity
  - Description of statistical methods (eg, complete description of fixed or random effects models, justification of whether the chosen models account for predictors of study results, dose-response models, or cumulative meta-analysis) in sufficient detail to be replicated
  - Provision of appropriate tables and graphics
- Reporting of results should include
  - Graphic summarizing individual study estimates and overall estimate
  - Table giving descriptive information for each study included
  - Results of sensitivity testing (eg, subgroup analysis)
  - Indication of statistical uncertainty of findings
- Reporting of discussion should include
  - Quantitative assessment of bias (eg, publication bias)
  - Justification for exclusion (eg, exclusion of non–English-language citations)
  - Assessment of quality of included studies
- Reporting of conclusions should include
  - Consideration of alternative explanations for observed results
  - Generalization of the conclusions (ie, appropriate for the data presented and within the domain of the literature review)
  - Guidelines for future research
  - Disclosure of funding source

 ©2000 American Medical Association. All rights reserved.

Discussion

The discussion should include issues related to bias, including publication bias, confounding, and quality. Bias can occur in the original studies (resulting from flaws in the study design that tend to distort the magnitude or direction of associations in the data) or from the way in which studies are selected for inclusion.[42] Publication bias, the selective publication of studies based on the magnitude (usually larger) and direction of their findings, represents a particular threat to the validity of meta-analysis of observational studies.[43-45] Thorough specifications of quality assessment can contribute to understanding some of the variations in the observational studies themselves. Methods should be used to aid in the detection of publication bias, eg, fail-safe procedures or funnel plots.[46]

Schlesselman[47] comments on such biases in assessing the possible association between endometrial cancer and oral contraceptives. This meta-analysis combined both cohort and case-control studies and used a sensitivity analysis to illustrate the influence of specific studies, such as those published in English.

Conclusion

Due to these biases in observational studies, the conclusion of the report should contain consideration of alternative explanations for observed results and appropriate generalizations of the conclusion. A carefully conducted meta-analysis can reveal areas warranting further research. Finally, since funding source has been shown to be an important source of heterogeneity,[48] the sponsoring organization should be disclosed and any effect on analysis should be examined.

COMMENT

Taking stock of what is known in any field involves reviewing the existing literature, summarizing it in appropriate ways, and exploring the implications of heterogeneity of population and study for heterogeneity of study results. Meta-analysis provides a systematic way of performing this research synthesis, while indicating when more research is necessary.

The application of formal meta-analytic methods to observational studies has been controversial.[42] One reason for this has been that potential biases in the original studies, relative to the biases in RCTs, make the calculation of a single summary estimate of effect of exposure potentially misleading. Similarly, the extreme diversity of study designs and populations in epidemiology makes the interpretation of simple summaries problematic, at best. In addition, methodologic issues related specifically to meta-analysis, such as publication bias, could have particular impact when combining results of observational studies.[44,47]

Despite these challenges, meta-analyses of observational studies continue to be one of the few methods for assessing efficacy and effectiveness and are being published in increasing numbers. Our goal is to improve the reporting of these meta-analyses so that readers can understand what was done in a given analysis, who did it, and why it was done. If bias is a problem, we suggest that an informative approach is to use broad inclusion criteria for studies and then to perform analyses (when the data permit) relating suspected sources of bias and variability to study findings.

Methodologic and interpretational concerns make the clear and thorough reporting of meta-analyses of observational studies absolutely essential. Our workshop was convened to address the problem of increasing diversity and variability that exist in reporting meta-analyses of observational studies. In constructing the checklist, we have attempted, where possible, to provide references to literature justifying the inclusion of particular items.

Assessment of the usefulness of recommendations for reporting is dependent on a well-designed and effectively conducted evaluation. The workshop participants proposed a 3-pronged approach to determine usefulness and implementation of these recommendations.

First, further comments should be incorporated into revisions of the checklist, to ensure its usefulness to journal reviewers and editors. The US Food and Drug Administration (FDA) receives and reviews petitions and applications for approval of regulated products and/or their labeling. The FDA's Center for Food Safety and Applied Nutrition is now receiving applications that use results of meta-analyses in support of the requested action. The revised checklist should be tested during the review of an application. One might randomly assign FDA reviewers who encounter systematic reviews of observational studies to use the checklist or not. Since the requirements for reporting for regulatory purposes might not completely coincide with those in the checklist and since sample size (the number of formal systematic reviews received by the FDA) might be small, this evaluation should document any potential incompatibility between requirements for regulatory reporting and the checklist.

Figure. Effect of Latent Period on Heterogeneity

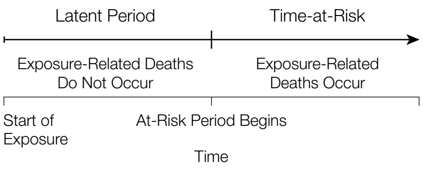

Second, we will work with the Cochrane Collaboration to promote the use of these recommendations by Cochrane collaborative review groups.[49] Members of the Cochrane Collaboration are involved routinely in performing systematic reviews. Some are now incorporating nonrandomized studies out of necessity. A trial of use of the checklist could be compared with the FDA experience.

Third, an evaluation of the checklist by authors, reviewers, readers, and editors could compare objective measures of the quality of articles written with and without the formal use of the guidelines. A challenge to the use of quality measures would be arriving at a valid measure of quality. A more important end point for trials in journals

©2000 American Medical Association. All rights reserved.

is process measures. Questions of interest include whether the use of the checklist makes preparation and evaluation of manuscripts easier or is otherwise helpful. Again, defining the constructs of interest present crucial challenges to this research.

Less formal evaluations, based on comments from users in any of the above groups, would certainly be helpful, as well. One would need to be concerned about contamination of the control groups when evaluating the checklist, as journals, for example, might adopt the checklist even in the absence of evidence of its efficacy from randomized trials.

In conclusion, the conference participants noted that meta-analyses are themselves observational studies, even when applied to RCTs.[50] If a role for meta-analyses of observational studies in setting policy is to be achieved,[51] standards of reporting must be maintained to allow proper evaluation of the quality and completeness of meta-analyses.

The Meta-analysis Of Observational Studies in Epidemiology (MOOSE) Group: Centers for Disease Control and Prevention, Atlanta, Ga: Gary Jeng, PhD, Rob Lyerla, PhD, Thomas Peterman, MD, Donna F. Stroup, PhD, MSc, Stephen B. Thacker, MD, MSc, and G. David Williamson, PhD; University of Pennsylvania School of Medicine, Philadelphia: Jesse A. Berlin, ScD; RAND Corporation, Santa Monica, Calif: Sally C. Morton, PhD; Stanford University, Stanford, Calif: Ingram Olkin, PhD; University of California, San Francisco, and *JAMA*, Chicago, Ill: Drummond Rennie, MD; Thomas C. Chalmers Centre for Systematic Reviews, Children's Hospital of Eastern Ontario Research Institute, Ottawa: David Moher, MSc; Michigan State University, East Lansing: Betsy J. Becker, PhD; Georgia State University, Atlanta: Theresa Ann Sipe, PhD; Centre for Statistics in Medicine, Oxford, England: Douglas Altman, PhD; Memorial Sloan-Kettering Cancer Center, New York, NY: Colin Begg, PhD; Hamilton Wentworth Regional Public Health Department, Hamilton, Ontario: Larry Chambers, PhD; Harvard Medical School and Brigham and Women's Hospital, Boston, Mass: Graham Colditz, PhD; University of Maryland, Baltimore: Kay Dickersin, PhD; AT&T Labs, Murray Hill, NJ: William DuMouchel, PhD; University of Colorado, Denver: Karen Kafadar, PhD; Cleveland, Ohio: Tom Lang, MA; Food and Drug Administration, Washington, DC: Lynn Larsen, PhD; University of Minnesota, Minneapolis: Thomas A. Louis, PhD; University of British Columbia, Vancouver: Parminder Raina, PhD; University of Pittsburgh, Pittsburgh, Pa: Allan Sampson, PhD; Family Health International, Research Triangle Park, NC: Ken Schulz, PhD, MSc; and Institute of Medicine, Washington, DC: Mike Stoto, PhD.

Acknowledgment: We thank Susan Eastwood, MA, University of California, San Francisco; Christine Friedenreich, PhD, Alberta Cancer Board and University of Calgary, Calgary; Sander Greenland, DrPH, Cstat, MA, MS, University of California, Los Angeles; Richard Horton, MBBCh, MD, *Lancet*, London, England; Diana Petitti, MD, MPH, Southern California Kaiser Permanente Medical Care Program, Pasadena; Duncan Saunders, MBBCh, PhD, University of Alberta, Edmonton; and Stephen D. Walter, PhD, McMaster University, Hamilton, Ontario, for their advice and substantive comments on an earlier draft of this article. In addition, we thank Barbara McDonnell, CDC, for managing complex logistics of this project.

REFERENCES

1. Greenland S. Quantitative methods in the review of epidemiologic literature. *Epidemiol Rev*. 1987;9:1-30.
2. Chalmers TC, Lau J. Meta-analytic stimulus for changes in clinical trials. *Stat Methods Med Res*. 1993; 2:161-172.
3. Badgett RG, O'Keefe M, Henderson MC. Using systematic reviews in clinical education. *Ann Intern Med*. 1997;126:886-891.
4. Ohlsson A. Systematic reviews. *Scand J Clin Lab Invest Suppl*. 1994;219:25-32.
5. Bero LA, Jadad AR. How consumers and policymakers can use systematic reviews for decision making. *Ann Intern Med*. 1997;127:37-42.
6. Thacker SB. Meta-analysis. *JAMA*. 1988;259:1685-1689.
7. Petitti D. *Meta-Analysis, Decision Analysis, and Cost Effectiveness Analysis.* New York, NY: Oxford University Press; 1994.
8. Berlin JA. Invited commentary. *Am J Epidemiol*. 1995;142:383-387.
9. Peipert JF, Phipps MG. Observational studies. *Clin Obstet Gynecol*. 1998;41:235-244.
10. Sipe TA, Curlette WL. A meta-synthesis of factors related to educational achievement. *Int J Educ Res*. 1997;25:583-598.
11. Ioannidis JP, Lau J. Pooling research results. *Jt Comm J Qual Improv*. 1999;25:462-469.
12. Lipsett M, Campleman S. Occupational exposure to diesel exhaust and lung cancer: a meta-analysis. *Am J Public Health*. 1999;89:1009-1017.
13. Vickers A, Cassileth B, Ernst E, et al. How should we research unconventional therapies? *Int J Technol Assess Health Care*. 1997;13:111-121.
14. Mann CC. Can meta-analysis make policy? *Science*. 1999;266:960-962.
15. Blettner M, Sauerbrei W, Schlehofer B, et al. Traditional reviews, meta-analyses and pooled analyses in epidemiology. *Int J Epidemiol*. 1999;28:1-9.
16. Greenland S. Invited commentary. *Am J Epidemiol*. 1994;140:290-296.
17. Lau J, Ioannidis JP, Schmid CH. Summing up evidence. *Lancet*. 1998;351:123-127.
18. Shapiro S. Meta-analysis/shmeta-analysis. *Am J Epidemiol*. 1994;140:771-778.
19. Stroup DF, Thacker SB, Olson CM, Glass RM. Characteristics of meta-analyses submitted to a medical journal. From: International Congress on Biomedical Peer Review and Global Communications; September 17-21, 1997; Prague, Czech Republic.
20. Lang TA, Secic M. *How to Report Statistics in Medicine.* Philadelphia, Pa: American College of Physicians; 1997.
21. Cook DJ, Sackett DL, Spitzer WO. Methodologic guidelines for systematic reviews of randomized control trials in health care from the Potsdam consultation on meta-analysis. *J Clin Epidemiol*. 1995; 48:167-171.
22. Moher D, Cook DJ, Eastwood S, et al. Improving the quality of reports of meta-analyses of randomised controlled trials. *Lancet*. 1999;354:1896-1900.
23. Huston P. Health services research. *CMAJ*. 1996; 155:1697-1702.
24. Egger M, Scheider M, Davey-Smith G. Meta-analysis. *BMJ*. 1998;316:140-144.
25. Stewart LA, Parmar MK. Meta-analysis of the literature or of individual patient data? *Lancet*. 1993; 341:418-422.
26. Steinberg K, Smith SF, Lee N, et al. Comparison of effect estimates from a meta-analysis of summary data from published studies and from a meta-analysis using individual patient data for ovarian cancer studies. *Am J Epidemiol*. 1997;145:1917-1925.
27. McManus RJ, Wilson S, Delaney BC, et al. Review of the usefulness of contacting other experts when conducting a literature search for systematic reviews. *BMJ*. 1998;317:1562-1563.
28. Hetherington J, Dickersin K, Chalmers I, Meinert CL. Retrospective and prospective identification of unpublished controlled trials. *Pediatrics*. 1989;84:374-380.
29. Cole MG, Bellavance F. Depression in elderly medical inpatients. *CMAJ*. 1997;157:1055-1060.
30. Kheifets LI, Afifi AA, Buffler PA, et al. Occupational electric and magnetic field exposure and leukemia. *J Occup Environ Med*. 1997;39:1074-1091.
31. Franceschi S, La Vecchia C, Talamini R. Oral contraceptives and cervical neoplasia. *Tumori*. 1986;72: 21-30.
32. Saag KG, Criswell LA, Sems KM, et al. Low-dose corticosteroids in rheumatoid arthritis. *Arthritis Rheum*. 1996;39:1818-1825.
33. Emerson JD, Burdick E, Hoaglin DC, et al. An empirical study of the possible relation of treatment differences to quality scores in controlled randomized clinical trials. *Control Clin Trials*. 1990;11:339-352.
34. Jüni P, Witschi A, Bloch R, Egger M. The hazards of scoring the quality of clinical trials for meta-analysis. *JAMA*. 1999;282:1054-1060.
35. Schulz KF, Chalmers I, Hayes RJ, Altman DG. Empirical evidence of bias. *JAMA*. 1995;273:408-412.
36. Berlin JA, for the University of Pennsylvania Meta-analysis Blinding Study Group. Does blinding of readers affect the results of meta-analyses? *Lancet*. 1997; 350:185-186.
37. Hasselblad V, Eddy DM, Kotchmar DJ. Synthesis of environmental evidence. *J Air Waste Manag Assoc*. 1992;42:662-671.
38. Friedenreich CM, Brant RF, Riboli E. Influence of methodologic factors in a pooled analysis of 13 case-control studies of colorectal cancer and dietary fiber. *Epidemiology*. 1994;5:66-67.
39. Berlin JA, Rennie D. Measuring the quality of trials. *JAMA*. 1999;282:1083-1085.
40. Colditz GA, Burdick E, Mosteller F. Heterogeneity in meta-analysis of data from epidemiologic studies. *Am J Epidemiol*. 1995;142:371-382.
41. Frumkin H, Berlin J. Asbestos exposure and gastrointestinal malignancy review and meta-analysis [published correction appears in *Am J Ind Med*. 1988; 14:493]. *Am J Ind Med*. 1988;14:79-95.
42. Blettner M, Sauerbrei W, Schlehofer B, et al. Traditional reviews, meta-analyses and pooled analyses in epidemiology. *Int J Epidemiol*. 1999;28:1-9.
43. Rosenthal R. The file drawer problem and tolerance for null results. *Psychol Bull*. 1979;86:638-641.
44. Easterbrook PJ, Berlin JA, Gopalan R, Matthews DR. Publication bias in clinical research. *Lancet*. 1991; 337:867-872.
45. Dickersin K, Min YI. NIH clinical trials and publication bias. *Online J Curr Clin Trials* [serial online]. 1993 Apr 28: Doc No 50.
46. Hedges LV, Olkin I. *Statistical Methods for Meta-analysis*. Boston, Mass: Academic Press; 1985.
47. Schlesselman JJ. Risk of endometrial cancer in relation to use of combined oral contraceptives. *Hum Reprod*. 1997;12:1851-1863.
48. Jadad A, Sullivan C, Luo D, et al. Patients' preferences for Turbuhaler or pressurized metered dose inhalers (pMDIs) in the treatment. From Annual Meeting of the American Academy of Allergy, Asthma, and Immunology; March 3-8, 2000; San Diego, Calif.
49. Huston P. Cochrane Collaboration helping unravel tangled web woven by international research. *CMAJ*. 1996;154:1389-1392.
50. Moher D, Pham B, Jones A, et al. Does the quality of reports of randomised trials affect estimates of intervention efficacy reported in meta-analyses? *Lancet*. 1998;352:609-613.
51. Berlin JA, Colditz GA. The role of meta-analysis in the regulatory process for foods, drugs, and devices. *JAMA*. 1999;281:830-834.

 ©2000 American Medical Association. All rights reserved.
